# Prolonged Sleep Apnea in Two Patients with a History of Opium Abuse -A Case Report

**DOI:** 10.22038/ijorl.2020.41832.2365

**Published:** 2020-03

**Authors:** Hadi Asadpour, Seyede Maryam Naghibi, Sadegh Rahimi, Amir Sharafkhaneh, Lahya Afshari Saleh, Fariborz Rezaee Talab, Mahnaz Amini, Faezeh Nikzad

**Affiliations:** 1 *Fellowship of Sleep Medicine, Psychiatry and Behavioral Sciences Research Center, Mashhad University of Medical Sciences, Mashhad, Iran.*; 2 *Psychiatrist, Psychiatry and Behavioral Sciences Research Center, Mashhad University of Medical Sciences, Mashhad, Iran.*; 3 *Neuroscience Research Center, Mashhad University of Medical Sciences, Mashhad, Iran, and Department of Pharmacology, Medical University Innsbruck, Innsbruck, Austria.*; 4 *Department of Medicine at Baylor College of Medicine, VAMC Sleep Center, Houston, TX.*; 5 *Occupational Medicine, Psychiatry and Behavioral Sciences Research Center, Mashhad University of Medical Sciences, Mashhad, Iran. *; 6 *Department of Neurological Diseases, School of Medicine, Mashhad University of Medical Sciences, Mashhad, Iran.*; 7 *Lung Disease Research Center, School of Medicine, Mashhad University of Medical Sciences, Mashhad, Iran.*

**Keywords:** Microarousal, Opium, Obstructive sleep apnea (OSA), Sleep Apnea, Sleep-disordered breathing (SDB)

## Abstract

**Introduction:**

Obstructive sleep apnea (OSA) is a highly prevalent sleep-disordered breathing (SDB).

**Case Report:**

Two 53- and 51-year-old male cases with daytime sleepiness and opium abuse and severe sleep apnea and long respiratory events duration (200 and 275 seconds respectively) noted in polysomnography were reported at Ebn-e-Sina and Razavi hospitals, in Mashhad, Iran. After positive airway pressure (PAP) therapy respiratory events resolved and patients’ daytime alertness improved.

**Conclusion:**

The long duration of sleep apnea could be the result of opium abuse. Therefore, drug history should be carefully considered in the evaluation of SDB patients. The PAP device was effective in the management of sleep respiratory events and the improvement of patient’s complications.

## Introduction

Obstructive sleep apnea (OSA) is a highly prevalent sleep-disordered breathing (SDB). The OSA is characterized by recurring episodes of upper airway obstruction during sleep. It may result in reduced airflow followed by a drop in oxygen saturation (i.e. hypopnea) or total cessation of breathing at least 10 sec (i.e. apnea) and lead to periodic arousal (i.e. short-time wakefulness during sleep) during sleep (1). The OSA is almost twice as prevalent in males compared to that reported for females (1). In addition, obesity has been known to be associated with OSA, and body mass index (BMI) positively correlates with the severity of the disease (1). Untimely diagnosis and proper treatment can lead to a range of serious complications, including increased systemic blood pressure, cardiovascular disease, cognitive impairment and psychiatric symptoms, such as depression, irritability, and anxiety (2). While daytime sleepiness and loud snoring are the main symptoms, a variety of complaints, including drowsiness, impaired cognition, fatigue and impatience, loss of attention and energy, morning headaches, and depressive symptoms, often constitute the clinical manifestations (2-4). Polysomnography (PSG) is the gold standard test for the diagnosis of SDB (5, 6). The main finding in PSG is the apnea-hypopnea index (AHI). The AHI is an index used to indicate the severity of sleep apnea, represented by the number of apnea and hypopnea events per hour of sleep. It is categorized as normal for the AHI <5, mild sleep apnea for 5 < AHI <15, moderate sleep apnea for 15 < AHI <30, and severe sleep apnea for AHI > 30 (3,4). 

It can be hypothesized that a higher number of respiratory events per hour of sleep is related to more complications. In a study conducted by Mediano et al., this hypothesis was supported that the patients with extreme daytime sleepiness experience a longer period of apnea and less oxygenation at the night (5); however, the role of the duration of apnea and depth of respiratory desaturation were neglected in the literature. The longer cessation of breathing coud cause a further decline in oxygenation, which leads to extreme stress to different organs of the human body; as a result, fatigue and daytime sleepiness also intensify. This study reported two cases of severe sleep apnea with a long duration of respiratory events, daytime sleepiness, and opium abuse.

## Case Report

Case 1 *:* A 53-year-old man with progressive daytime sleepiness and disrupted sleep underwent split-night PSG at the sleep laboratory of Ebn-e-Sina hospital, in Mashhad, Iran. He was treated with lorazepam 2 mg at night in the last 3 months with no improvement in sleep patterns. Over the last 2 years, he complained of vague headaches, as well as feeling depressed and impatient. The patient was overweight (BMI: 28.4). He was also diagnosed with hypertension and hypercholesterolemia since 2 years ago. The drug history included the administration of atorvastatin 20 mg daily and captopril 25 mg twice a day. In the clinical examination, blood pressure was 140/85 mmHg. He was heavy smoker (120 packs/year) and reported a history of opium abuse for 10 years. He smoked 3-4 gr traditional opium at home every day. The Epworth Sleepiness Scale (ESS) was 20 (out of maximum 24) indicating severe subjective daytime sleepiness. The STOP-BANG score was 7 (out of 8) indicative of a severe respiratory interruption during sleep. The Min-Mental Status Examination score was 29. On the Hamilton Depression Rating Scale (HAM-D), he had a score of 30 consistent with major depression. During the previous 5 months, he was under treatment for depression with sertraline 150 mg twice a day with no improvement in his mood. 

The patient’s demographic characteristics and questionnaire scores is described in (Table.1). 

**Table 1:** Demographic characteristics and the questionnaires scores of two patients

	**Age**	**BMI**	**ESS**	**STOP-BANG**	**neck circumference**
	53	28.4	20	7	45
	51	35.3	12	8	48

AI: Arousal index, AHI: Apnea-hypopnea index, BMI: Body mass index, ESS: Epworth Sleepiness Scale,

He underwent an attended Split-Night PSG (by Harmonia software version 7) with concomitant video recording that was performed according to the guidelines of the American Academy of Sleep Medicine (AASM) with simultaneous monitoring of electroencephalography (EEG), electrooculo- graphy (EOG), Chin electromyography (EMG), airflow and respiratory efforts, pulse oximetry, electrocardiography (EKG), and limb EMG. The scoring manual (version 2.2) of the AASM was used for scoring the sleep study (6).

Overnight PSG demonstrated an AHI of 110.8 per hour of sleep compatible with severe sleep apnea. The notable finding was an obstructive apnea episode lasted for 3 minutes and 20 seconds during the rapid eye movement (REM) period accompanied by electrocardiography(EKG) abnormality and cyanosis, and it was terminated by an awakening.

The mean oxygen saturation was 88.2%. The minimum oxygen concentration was lower than 55.9%. The desaturation index was 146 per hour of sleep. Arousal occurred with a total arousal index of 88.1, mainly due to respiratory events. ECG demonstrated frequent transient ST elevation and arrhythmia during oxygen desaturation. The patient underwent titration through Bilevel Positive Airway Pressure (BPAP) with the optimal response at BPAP of EPAP=13 cmH_2_O and IPAP=17 cmH_2_O. After 1 month of OSA management by BPAP, sleep indices improved which is summarized (Table.2).

**Table 2:** Polysomnography indices of two patients before titration study

	**N1%**	**N2%**	**N3%**	**REM%**	**Sleep period (min)**	**Sleep efficiency%**	**AI**	**AHI**	**Mean duration (sec)**	**Longest duration (sec)**	**ODI**	**Ds< 90%**	**Mean SpO2 (%)**	**Lowest Sp O2 (%)**	**PLMI**
	18	78	0	4	72	49	88	111	15.8	200.4	36	59.3	94	< 50	9
	32	58	0	10	226	90.6	42	62.7	22.1	275.6	89	99.2	79.6	50	4.4

AI: Arousal index, AHI: Apnea-hypopnea index, , REM: Rapid eye movement, ODI: Oxygen desaturation events index, **Ds: **Desaturation, PLMI: Periodic leg movement index

In the following months, he was visited several times. He was satisfied with the permanent application of BPAP.

In addition, he was reported with less daytime sleepiness and depression-related symptoms (The HAM-D score decreased to 14). 

Case 2 *: *A 51-year-old man complained of nightly snoring and daytime tiredness, exacerbated by gaining weight since 12 years ago, breathing pauses, and waking up gasping for air. The sleep was reported as restless and unrefreshing. In the last month, he tried several sedative drugs, such as diphenhydramine 10 mg or lorazepam 1 mg, through the mouth with no improvement. Morning headaches, feeling sad, boredom, drowsiness, and attention deficit were his complaints throughout the day.

 The patient’s demographic characteristics and questionnaire scores is described in Table 1. In the past medical history, he had elevated blood pressure; however, he was not on any pharmacotherapy. He smoked cigarette (50 packs/year) and used opium in the form of buprenorphine (B2). He also had polycythemia (Hct: 62%). Venous blood gas prior to PSG showed hypercapnia with PCO2 of 62 mmHg and HCO3 of 31 mg/dl. He was admitted to the sleep center of Razavi Hospital and underwent split-night PSG (by Remlogic software version 3.2) for 8 h at night. The sleep time during this study was 5 h and 15 min during which 216 occasions of respiratory disturbances, including apneas and hypopneas, occurred with an AHI of 62.7 indicative of severe OSA. The results of the study showed several prolonged obstructive apnea episodes during the REM with a maximum duration of 4 min and 35 seconds. The event was terminated with patient awakening. The oxygen saturation was 79.6% on average. 

The minimum oxygen saturation was 50%. The blood oxygen desaturation index was 89.4 during sleep. In 45.2% of sleep duration, he had loud snoring. The EKG demonstrated arrhythmias, including wide narrow complex tachycardia and bradycardia (as low as 34 beats per minute) and tachycardia (up to 132 beats per minute). 

Table 3 shows the main PSG indices of the two patients. The patient was titrated with BPAP (IPAP of 22 and EPAP: 18 cm H2O). Sleep indices (including oxygen saturation and arousal index) improved which is summarized in table 2. He was recommended to be evaluated regarding other possible omorbidities, especially through phlebotomy; however, he refused and left the study.

**Table 3:** Polysomnography indices of two patients after titration study

	**N1 %**	**N2 %**	**N3 %**	**REM %**	**Sleep period (min)**	**Sleep efficiency%**	**AI**	**AHI**	**Mean duration (sec)**	**Longest duration (sec)**	**ODI**	**Ds< 90%**	**Mean SpO2 (%)**	**Lowest Sp O2 (%)**
	17	39	13	31	259	64	22	25	34	173.2	21	59.3	90	60
	7.5	37	15	45	239	98	9	3.8	30	174	15	99.2	85.4	51

AI: Arousal index, AHI: Apnea-hypopnea index, REM: Rapid eye movement, ODI: Oxygen desaturation events index **Ds: **Desaturation

**Case 1:**
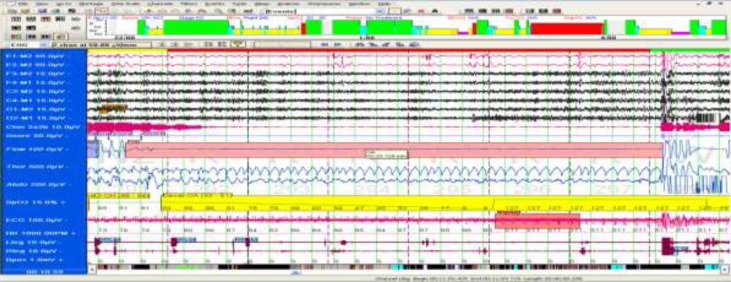
4 min. epoch N2 REM stage sleep with Snoring, Obstructive Apnea (3 minutes and 20 seconds), with Arousal, Desaturation & RRLM

**Case 2:**
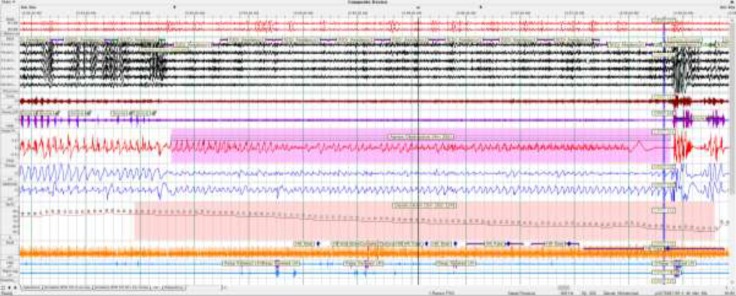
Pretreatment 5 min. epoch REM sleep stage with snoring, Obstructive Apnea (4 minutes and 35 seconds) with Arousal, Desaturation, wide complex tachycardia and Respiratory related leg movements

## Conclusion

The authors noticed long sleep apnea events in two patients complaining of snoring, daytime sleepiness, tiredness, and sleep disturbance. On PSG, the duration of two respiratory events were 200 and 275 seconds. The apnea durations observed for the two cases are the longest reported in Iran and among the longest in the world (7). The AHI were 110.8 and 62.7 for the first and second cases, respectively, which revealed very severe sleep apnea in the patients. Due to the extreme duration of the single apnea event and very high AHI, these cases were worthwhile to report. 

The unusual duration of the single apnea event, as well as the AHI, might be caused by various risk factors, including obesity or depression. However, these factors are frequently observed in patients, suffering from mild to severe OSA, no similar studies found such remarkable figures. In addition, these reported cases had no central apnea, and neurologic examination was normal. Accordingly, opium abuse was considered the root of this notable sleep disturbance in both patients. The opioid-specific receptor, μ (mu), is widespread at various neuronal sites controlling the human breathing. Opioids descend intracellular cyclic AMP levels and suppress respiratory neuronal function via μ receptors (8). Nevertheless, most studies identified central sleep apnea, mixed sleep apnea, and OSA in patients with opium abuse, some studies reported that OSA is more prevalent in chronic opium abuse (9). Opioids can reduce genioglossus muscle activity, which can predispose the patient to upper airway closure and severe OSA (8). 

All the respiratory events were of obstructive type and the majority occurred during REM sleep stage. During REM period, the oropharyngial muscles’ tone decreased and simultaneously the arousal threshold increased. These physiologic changes would be exacerbated with opium use. (8,9) This is the probable mechanism explains the prolonged sleep apnea in our patients. Micro-arousals, preventing longer hypoxia during sleep, might be suppressed by the central effect of opium. Therefore, chronic opium abuse can cause a longer duration of single apnea events in due to lack of arousal. Despite opium abuse, resulting in significant apnea duration, both patients who benefited from PAP therapy revealed its effective role in the management of the diverse range of OSA. However, the impact of opium withdrawal on sleep architecture should be precisely evaluated in further studies. 

PAP device is undoubtedly the gold standard treatment for OSA. The PAP is highly effective in controlling symptoms, improving quality of life, and decreasing the clinical complications of sleep apnea similar to the number of nocturnal obstructive events and nocturnal arousals, improving sleep parameters and nocturnal oxygen saturation from the start of treatment (10).

 In fact, effective treatment requires long-term management. The careful monitoring of the subject and increased patient adherence to treatment, providing a variety of therapeutic alternatives, as well as the appropriate treatment for comorbidities and associated disorders, will help to achieve the best results. It seems that the efficacy of PAP might be limited by suboptimal adherence. 

The long duration of sleep apnea could be the result of opium abuse; therefore, drug and habitual history should be carefully considered in the SDB approach. The PAP device was effective in the management of sleep apnea, despite the high AHI and concomitant depression, obesity, and drug abuse.
